# Epistemic trust, mentalizing and extremist attitudes in a German community sample

**DOI:** 10.3389/fpsyt.2026.1934135

**Published:** 2026-10-20

**Authors:** Sophie Hauschild, Anna Berning, Kaine Heimert, Lea A. Kasper, Svenja Taubner

**Affiliations:** 1 Institute for Psychosocial Prevention and Psychotherapy, University Hospital Heidelberg, Heidelberg, Germany; 2 Deutsches Zentrum Psychische Gesundheit (DZPG), Heidelberg, Germany

**Keywords:** epistemic mistrust, epistemic trust, extremist attitudes, gender, mental disorder, mentalizing, mentalization

## Abstract

**Introduction:**

While extremist attitudes, here conspiracy mentality, antisemitic attitudes, and the ideological support of violence, are typically studied in political or sociological psychological contexts, their investigation in relation to clinical psychological concepts may offer additional insight with respect to underlying psychological mechanisms. In this study, we aimed to explore the relation between self-reported extremist attitudes and epistemic trust, mistrust, and credulity, as well as mentalizing. The different epistemic stances as well as difficulties in the ability to mentalize are assumed to represent non-pathological individual variations in psychological vulnerability and resilience.

**Methods:**

This questionnaire study investigates the relationship between levels of epistemic trust, mistrust, and credulity, assessed via the Epistemic Trust, Mistrust and Credulity Questionnaire, mentalizing deficits assessed via the Mentalization Questionnaire and endorsement of extremist attitudes (antisemitic attitudes assessed via 2 items of the model questionnaire on right-wing extremism by the German Federal Agency for Civic Education, conspiracy mentality assessed via the Conspiracy Mentality Questionnaire, and violence supporting attitudes assessed via the Violent Extremist Attitudes Scale) in a German community sample (N = 566). Due to scarcity in empirical research on these concepts, we took an exploratory approach to investigate potential associations using structural equation modeling. Self-indicated presence of a mental disorder and participants` gender were integrated into the model.

**Results:**

Epistemic mistrust and all extremist attitudes were positively related via mentalizing deficits; as was the marginally significant negative association between epistemic trust and conspiracy mentality. No other indirect effects of epistemic trust or credulity on extremist attitudes reached significance. Presence of a mental disorder was associated with epistemic credulity, epistemic mistrust, and mentalizing deficits, but not with extremist attitudes. Female gender was associated with higher epistemic trust and lower antisemitic attitudes as well as lower endorsement of violence supporting attitudes.

**Conclusion:**

Exploratory findings should inspire future longitudinal or experimental research on the investigated constructs to identify whether they might be promising candidates to reduce extremist attitudes in the general population. Moreover, gender-specific mechanisms seem of relevance and need to be further explored.

## Introduction

The rise of extremist attitudes has become a central topic in societal and political discourse. Attitudes are commonly defined as relatively stable evaluative orientations toward an object, person, or idea, comprising cognitive, affective, and behavioral components (1). Extremist attitudes represent a specific, ideologically charged subset of this general construct, including attitudes, behaviors, organizations and goals that oppose the democratic state (2). Kingdon (3) describes extremist attitudes as a broad scale of behaviors reaching from strong political views (e.g., right- and left-wing extremism) to support and enactment of physical violence that challenge the democratic norms. In Germany, recent reports indicate an increasing endorsement of extremist attitudes within the general population (4). Extremist attitudes cannot be seen as a unitary phenomenon but encompass different, though often interrelated, belief systems. In this study, we focus on conspiracy mentality, antisemitic attitudes and violence supporting attitudes in a German community sample. Conspiracy theories are commonly defined as beliefs that powerful and malevolent groups operate in secrecy to achieve harmful goals (5). Conspiracy mentality can be described as an individual´s tendency to believe in such theories (6). Antisemitism, though lacking a universally accepted definition, is broadly conceptualized as a worldview which attributes societal, political, and cultural problems to the existence of Jewish people, accompanied by affective hostility, stereotyping, and delegitimization of Jews (7). Violent extremist attitudes, as investigated here, refer to the ideological justification of violence to achieve political, religious, or social goals (8).

These belief systems are closely intertwined: For example, conspiracy thinking has been identified as a robust predictor of antisemitic behavior (9). A recent study by Allington et al. (10) found significant positive associations between various forms of antisemitism (e.g., prejudicial attitudes toward Jews and Israel-related bias) and endorsement of conspiracy beliefs (e.g., malevolent global conspiracies, control of information). In a series of exploratory experiments conducted by Lewis (11), strong associations were found between conspiracy and antisemitic views, which in their specifics depended on political orientation of the participants. In a German representative study, conspiracy mentality predicted increased violent extremist attitudes (12). As observed by Vegetti et al. (13), conspiracy belief significantly predicted the justification of political violence and was also the basis for legitimizing radicalism. Together, these findings indicate that conspiracy mentality, antisemitic attitudes, and violence-supporting attitudes are empirically linked rather than independent phenomena.

Psychological research on mechanisms contributing to extremist views has increasingly highlighted the relevance of aversive emotional experiences in the formation of extremist beliefs: For example, fear experienced during the COVID-19 pandemic was linked to heightened authoritarian attitudes (14). Moncrieff and Lianard (15) suggested that a strong desire to correct perceived injustices – particularly when unresolved – can motivate extremist worldviews, including support for violence. Chronic exposure to disadvantage and discrimination [e.g., structural exclusion or humiliation (16)] has also been linked to increased endorsement of extremist attitudes. However conversely, it is also possible that a pre-existing tendency toward aggression or norm-violation is retrospectively legitimized through extremist ideologies (17). From a motivational perspective, Kruglanski et al. (18) proposed that extremism may arise from a chronic imbalance in which one fundamental need (e.g., control) dominates at the expense of others (e.g., relatedness). Additionally, gender-related aspects are considered increasingly important. Gender is understood as a range of socially constructed roles, attitudes, and behaviors of being a man, a woman, or self-defined third option (e.g., non-binary), and is distinct from biological sex [e.g., being female; (19)]. According to the literature, men score higher on dimensions (e.g., permissibility of aggression) of violent extremism than women (20, 21).

Thus, although extremist beliefs are often accompanied by suffering and social dysfunction, they are not indicative of mental illness. Yet, the application of concepts used in psychological clinical research might provide valuable insight as they shed light on normal psychological mechanisms occurring when people are in distress. For example, in a recent meta-analysis (22) exploring the findings of 40 studies, significant associations have been observed between conspiracy thinking and clinical traits such as narcissism and paranoia; moreover, associations were proposed to be mediated by disrupted epistemic trust (23, 24). Epistemic trust is a nonpathological attribute which refers to a communicational stance that allows to flexibly suppress natural epistemic vigilance when it is warranted and consider socially transmitted information as relevant, trustworthy, and reliable (25, 26). Disruptions of epistemic trust as communicational stances – namely epistemic mistrust, involving interpersonal hypervigilance, or epistemic credulity, including indiscriminate trust in unreliable sources – may develop in the context of adverse early interpersonal experiences and are increasingly studied in both clinical psychology as a nonpathological transdiagnostic vulnerability (27) as well as in their relationship to conspiracy mentality: both epistemic mistrust [e.g. (28)] and epistemic trust have been positively linked with conspiracy mentality (29). The latter was found by our workgroup as an indirect link via increased paranoid distress measured as how distressing individuals would find interpersonally threatening contexts. As individuals high in epistemic trust tend to rely on others for information, the authors concluded that epistemic trust might come along with a vulnerability to engage in conspiracy belief in the context of an (imagined) threatening interpersonal environment due to disrupted regulation strategies. Additionally, an individual’s epistemic stance is assumed to be closely intertwined with their ability to mentalize and both serve self- and interpersonal regulatory and adaptive functions (29). Mentalizing is the ability to interpret one’s own and others’ behaviors in terms of underlying mental states [e.g., beliefs, desires, intentions; e.g. (30)]. Deficits in mentalizing appear when stress is high and impair one’s ability to accurately evaluate social information, coming along with an increased risk of rigid, distorted beliefs (25, 31, 32). In line, effective mentalizing was proposed to come along with increased epistemic trust (25), while epistemic mistrust and credulity combined with mentalizing impairments, were associated with self- and interpersonal difficulties and reduced adaptation to society (33). Mentalizing difficulties and trust disturbances have also been associated with gender, with women showing higher epistemic trust, credulity, and an advantage in mentalizing abilities, while men showing higher epistemic mistrust and lesser degrees of mentalizing abilities (34, 35).

Epistemic trust, epistemic mistrust, and credulity, as well as mentalizing represent clinically grounded yet nonpathological and dimensional psychological constructs that may offer valuable insights into the emergence and maintenance of extremist thinking within the general population. As these concepts have received limited empirical attention outside of the clinical domain so far, and the empirical associations found point to complexity of relationships, the present study aims to explore possible associations between epistemic stance (trust, mistrust, and credulity), mentalizing, and extremist attitudes, including antisemitic beliefs, conspiracy ideation, and violent extremism, in a German community sample as a first step to extend explorations to a broader adult population in Germany. Psychiatric diagnoses and gender are also included as potential confounders in the analyses.

### Hypotheses

H1: All aspects of epistemic stance (trust, mistrust, credulity) are associated with deficits in the ability to mentalize.
H2: Deficits in the ability to mentalize are associated with the extent of supporting extremist views in a German community sample (antisemitic attitudes, conspiracy mentality, and violence supporting attitudes).
H3: There are indirect effects between all aspects of epistemic stance and the extent of support of extremist views via mentalizing deficits.

## Methods

### Participants

German community participants were recruited via the panel provider Respondi. Inclusion criteria were 1) sufficient German language proficiency, 2) an age between 18 and 65 years. No additional exclusion criteria were applied, as the aim of the study was to examine individual differences in epistemic trust, mentalizing, and extremist attitudes in a community sample. A total of 1, 101 individuals participated in the online survey. The present study partly represents a partly secondary analysis of data originally collected for the validation of a questionnaire (36). Consequently, no *a priori* power analysis was conducted for the specific structural equation model examined in the present study. However, a RMSEA-based sensitivity analysis (37) indicated excellent statistical power (>.99) for the estimated structural equation model. The final sample size exceeded commonly cited recommendations for structural equation modeling (37). Following the predefined data cleaning procedure (see Data Analysis), the final sample comprised *N* = 566 participants. The final sample had a mean age of 43.25 years (SD = 13.88; range = 18–65 years). 49.12% of the participants identified as female, 50.35% as male, and 0.53% as diverse (individuals who do not want to and/or are not able to assign themselves to the binary system male/female). Based on self-report, 25.1% of participants reported having experienced a mental disorder within the previous year. Further sociodemographic data are presented in **Table 1**.

**Table 1:** Sociodemographic data (N = 566).

Variable	n	(%)	M	(SD)
Gender				
Female	278	(49.1)		
Male	285	(50.4)		
Diverse*	3	(0.5)		
Age			43.25	(13.9)
Highest educational attainment				
Currently in school	2	(0.4)		
Middle school diploma	89	(15.7)		
High school diploma	116	(20.5)		
Professional apprenticeship	166	(29.3)		
Bachelor’s degree	74	(13.1)		
Master’s degree	114	(20.1)		
PhD	5	(0.9)		
Current employment				
Student in school	4	(0.7)		
In training	9	(1.6)		
Student in University/College	51	(9.0)		
Employee	356	(62.9)		
Self-employed	39	(6.9)		
Unemployed/job-seeking	70	(12.4)		
Civil servant	29	(5.1)		
Missing	8	(1.4)		
Mental disorder within past year				
Yes	142	(25.1)		
No	424	(74.9)		

M, arithmetic mean; SD, standard deviation; n, number of participants.

* Refers to individuals who do not want to and/or are not able to assign themselves to the binary system male/female.

### Procedure

The German online survey was conducted in February 2022 via SoSci Survey (38). The recruitment was carried out via the panel provider Respondi, which is a pure market research panel with voluntary membership and participation via double opt-in. Each participant was permitted to take part in the study only once and was compensated by Respondi in accordance with the pre-established survey duration. The measures addressing our hypothesis were part of a questionnaire package designed to take 45 minutes to complete. The questionnaire package additionally contained measures that were not relevant to the present secondary analyses (36). The study was approved by the Ethics committee of the Faculty of Behavioral and Cultural Studies at the University of Heidelberg (AZ Tau 2020 1/1).

### Measures

The Epistemic Trust, Mistrust, and Credulity Questionnaire (ETMCQ) (27), German version by Weiland et al. (39), is a self-report questionnaire, assessing epistemic trust (5 items), mistrust (3 items) and credulity (4 items). The 12-item version represents a revised and shortened form of the original 15-item questionnaire. Items (e.g., “I’d prefer to find things out for myself on the internet rather than asking people for information”) are rated on a seven-point Likert scale from “strongly agree” (1) to “strongly disagree” (7). The internal consistencies of the subscales in the current study were α=.77 for epistemic trust, α=.60 for epistemic mistrust and α=.78 for epistemic credulity.

The Mentalization Questionnaire (MZQ) (40) is a 15 item self-report questionnaire that assesses deficits in the ability to mentalize. The items (e.g., “If someone yawns in my presence, that’s a reliable sign that he is bored in my company.” or “Often I don’t even know what is happening inside of me.”) focus on different aspects of mentalizing impairment such as deficits in emotional awareness, self-reflection or emotion regulation. Items are rated on a five-point Likert scale ranging from “no agreement at all” (1) to “total agreement” (5), with higher score indicating greater deficits in the ability to mentalize, i.e., lower mentalizing ability. The MZQ shows good internal consistency in previous research [ω = 0.88; (41)] as well as in the current study (α = .87).

The Conspiracy Mentality Questionnaire (CMQ) (6) is a 5-item self-report questionnaire, assessing the tendency to engage in conspiracy thinking (e.g., “I think that there are secret organizations that greatly influence political decisions”). Answers can be given on an 11-point scale ranging from 0 (0% - certainly not) to 10 (100% - certain). The German version of the CMQ showed good results in former studies [α = .84; (6)] as well as in the current study (α = .88).

The Violent Extremist Attitudes Scale (Gewaltbereite Extremistische Einstellungen, GEE) (8) is a 4-item self-report questionnaire assessing the endorsement of violence supporting attitudes. Items (e.g., “It’s sometimes necessary to use violence to fight against things that are very unjust”) consists of statements agreeing with violent supporting attitudes and can be rated on a 4-point Likert scale ranging from “fully untrue” (1) to “fully true” (4). The internal consistency in the current study was α = .90.

Questions regarding antisemitic attitudes were extracted from the adapted version of the model questionnaire on right-wing extremism (42). Two items were used to assess antisemitic attitudes (“The Jewish people determine the international financial markets” and “The Jewish people use the crimes committed by the Nazis against the Jewish people in WW2 to create a better position for themselves within society”). The items were rated on a five-point Likert scale from “strongly agree” (1) to “strongly disagree” (5), i.e., higher scores indicating lower antisemitic attitudes. In order to facilitate the comprehension of the hypothesis formation process, the sum score was inverted so that higher values correspond to a higher level of agreement with antisemitic thinking. Due to the limited number of items included on the test, an evaluation of internal consistency was conducted using the Spearman-Brown Coefficient. The results indicated a good level of consistency (ρ=.87).

Sociodemographic information was assessed via self-report and included age, gender, educational attainment, current employment status, and whether participants had experienced a mental disorder within the previous year (yes/no).

### Data analysis

Data analysis was performed using the desktop version of R-Studio (43). To ensure data quality, two instructed response items were implemented at two timepoints of the survey (e.g., “If you are attentive, please answer very much.”). *n* = 483 participants answered the items incorrectly, reducing the total *N* = 1101 to a subsample of *n* = 618. Further data cleaning steps were divided into two aspects of response anomalies of careless responders (44). The initial step entailed the exclusion of participants whose response times were excessively fast as indicated by the SoSci-generated variable “Time_RSI”, which reflects deviations in response time from the median (45). Participants with a Time_RSI value above the predefined cutoff of 2 were classified as excessively fast responders and excluded from further analyses (*n* = 50). Subsequently, the sample was examined for any contradictory responses to items within the ETMCQ. Given the conceptual distinction between epistemic trust, mistrust, and credulity, answering all items with the same extreme response category (1 or 7) was considered indicative of an invalid response pattern. A total of *n* = 2 was excluded at this stage of the process. The final sample comprised 566 participants and contained no missing values for the central study variables. Furthermore, the central constructs of the analyses were examined with regard to the measures of dispersion and location of the distribution, as well as multiple outliers using Mahalanobis distance. Cases exceeding the critical χ² value (p <.001) were considered potential multivariate outliers. Three participants met this criterion. Sensitivity analyses excluding these participants yielded no meaningful differences in the pattern of correlations; therefore, the cases were retained. The decision to include gender as a control variable resulted in the necessity to consider the varying group sizes of the gender categories. Due to the discrepancy in sample size between male (*n* = 285) and female (*n* = 278) participants, as well as non-binary individuals (*n* = 3), it was decided that non-binary participants would be excluded from the structural equation model calculation. This decision was made to ensure meaningful statistical estimations. Nevertheless, the non-binary sample was included in the descriptive statistics.

For preparatory analyses intercorrelation and measurement models of the central constructs were calculated. A structural equation model (SEM) was then run to test the hypotheses. According to the hypotheses the model included 3 independent variables (stances of epistemic trust), one mediating variable (impairments in the ability to mentalize) and three dependent variables (endorsement of violence supporting attitudes, conspiracy thinking, antisemitic attitudes). Accordingly, the model included a total of 9 indirect pathways that were analyzed (model is depicted in **Figure 1**). Given that n=142 participants reported experiencing a mental disorder within the past year, it was decided to include the variable mental disorder (yes/no) as a dummy-coded control variable. Additionally, gender was selected as a dummy-coded control variable. This approach ensured that the effects examined could be estimated independently of gender differences and that any potential distortions could be identified. The model was evaluated using model fit indices. The Comparison Fit Index (CFI) indicates an acceptable with values ≥.9. The Root Mean Square Error of Approximation (RMSEA) as well as the Standardized Root Mean Square Residual (SRMR) demonstrate acceptable model fits with values ≤.08 (46). Following the initial fit assessment of the hypotheses testing model, content coherent intercorrelations were permitted in accordance with the R-Studio algorithm (47).

**Figure 1:**
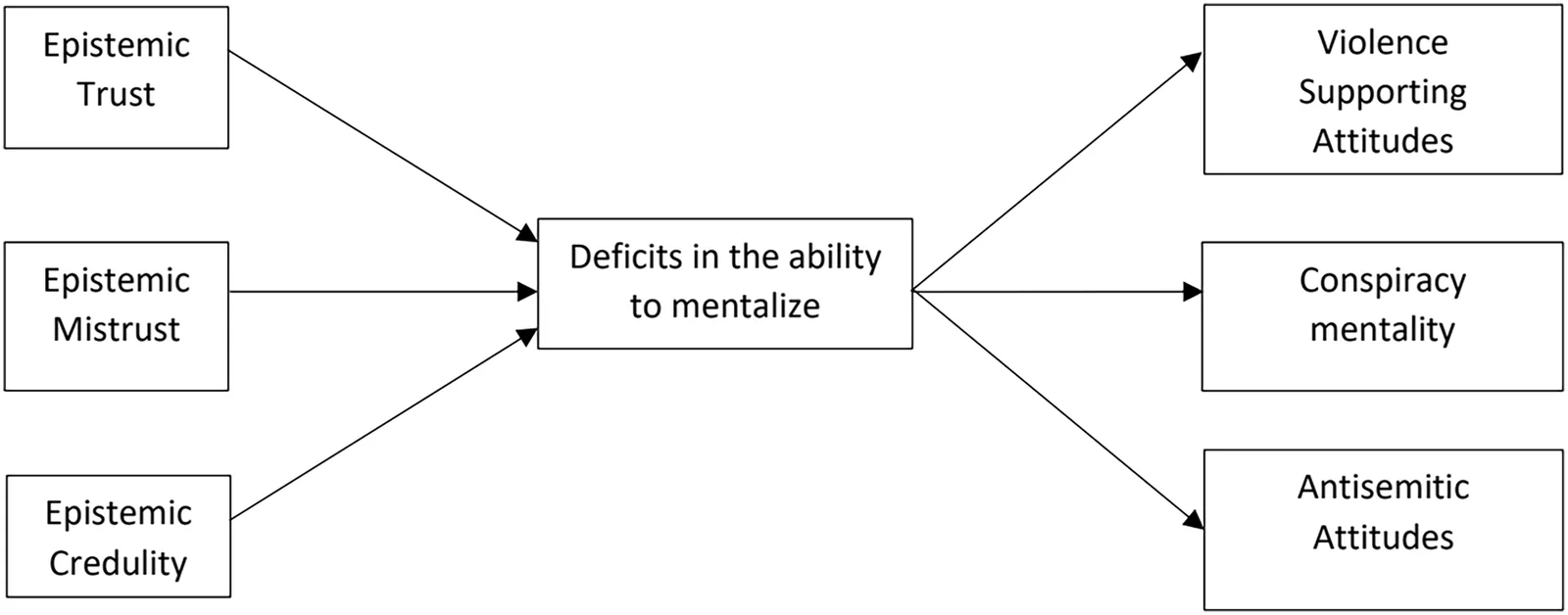
Graphic illustration of hypotheses.

## Results

### Descriptive and preparatory analyses

The inter-construct correlations as well as the descriptive values of the central constructs are shown in **Table 2**. Contrary to the expectations, epistemic trust did not correlate with epistemic mistrust and all forms of extremist attitudes. Credulity and the endorsement of violence supporting attitudes showed no significant association. Cronbach’s alpha coefficients indicated acceptable to good internal consistency for all assessed constructs (α=.77-.90), with the exception of epistemic mistrust. The epistemic mistrust scale demonstrated questionable internal consistency (α = .6), which is likely attributable to the small number of items (three). This limitation was considered when interpreting the results. In order to conduct preparatory analyses, the measurement models were evaluated using the subsequent model fit indices. Once the theory-compliant intercorrelations had been allowed for, all measurement models demonstrated an adequate fit in the overall view of the indices (CFI ≥.905; GFI ≥.919; RSMEA ≤.188; SRMR ≤.061). The normal distribution of the central constructs was examined via skewness and kurtosis values, following the rule of thumb set forth by Hair et al. (48), as well as through visual inspection. All variables exhibited acceptable skewness and kurtosis values. However, the visual inspection of the endorsement of antisemitic attitudes and violence supporting attitudes indicated that they may not be normally distributed. In light of these considerations and the use of two categorical control variables, the WLSMV estimator was deemed the most appropriate.

**Table 2:** Means, standard deviations, correlations and internal consistencies (Cronbach’s alpha).

Variable	M	SD	1	2	3	4	5	6	7
1. ETMCQ-Trust	23.33	5.38	(.77)						
2. ETMCQ-Mistrust	14.59	4.34	-.044	(.60)					
3. ETMCQ-Credulity	14.90	6.09	.081*	.52***	(.78)				
4. MZQ	39.44	10.40	-.14**	.54***	.49***	(.87)			
5. GEE	6.20	2.82	-.03	.12**	.05	.12***	(.88)		
6. CMQ	25.79	13.02	.05	.32***	.23***	.22**	.19***	(.90)	
7. Antisemitic attitudes	1.59	2.24	-.05	.13**	.11*	.10*	.21***	.47***	(.87)^a^

M, arithmetic mean; SD, standard deviation; ETMCQ, Epistemic Trust; Mistrust, and Credulity Questionnaire; MZQ, Mentalization Questionnaire; GEE, Violent Extremist Attitudes Scale; CMQ, Conspiracy Mentality Questionnaire.

***p≤.001; **p≤.01, *p≤.05.

^a^ calculated with spearman-brown coefficient due to small item number.

### Hypotheses testing

With regard to the hypothesis testing model, all indices demonstrated an acceptable fit, as evidenced by the following values: CFI = .944; RMSEA = .046; SRMR = .058. A comprehensive graphical representation of the model with all regression coefficients can be obtained from **Figure 2**. The following section presents the results in accordance with the aforementioned hypotheses.

**Figure 2:**
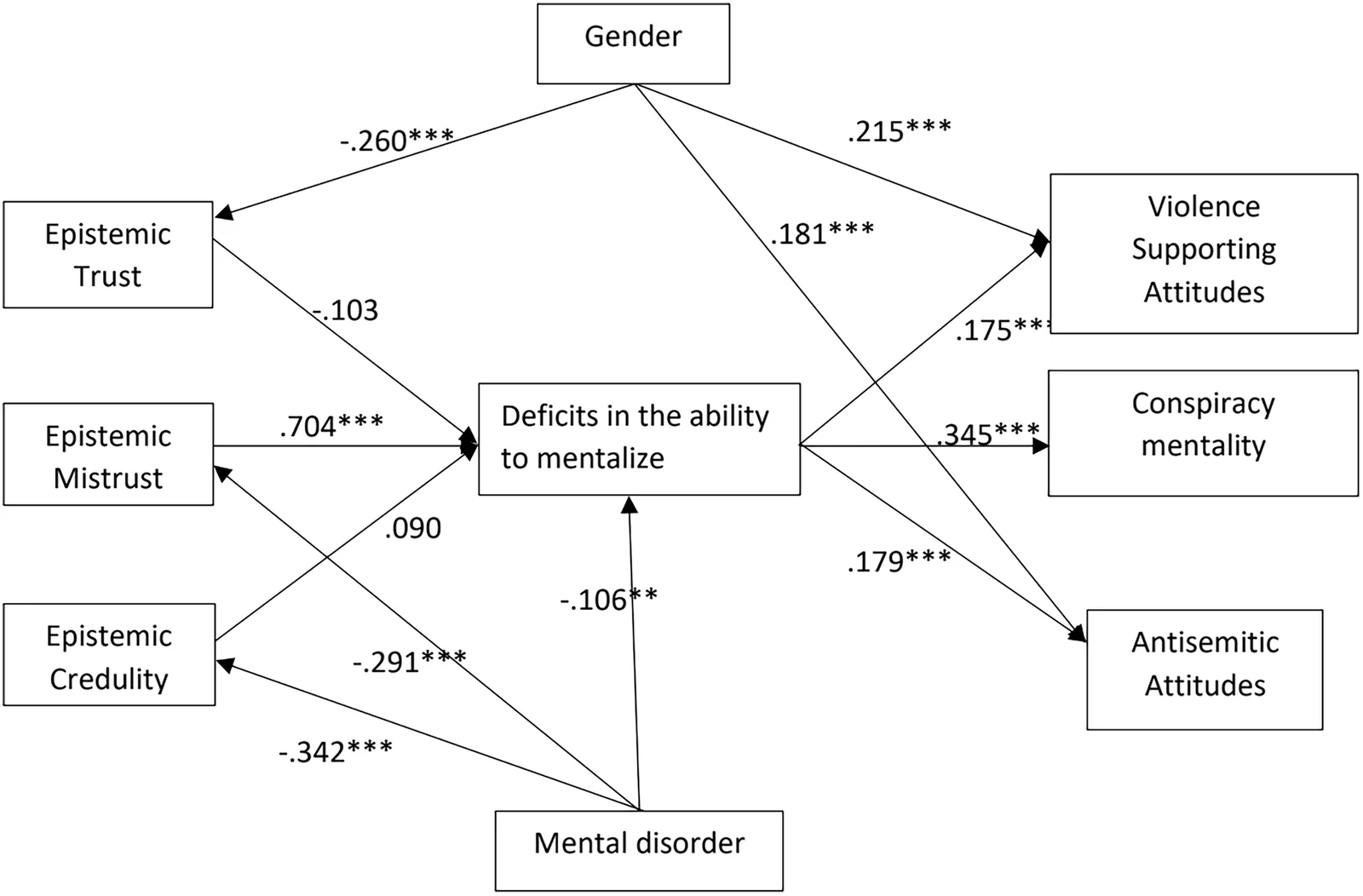
SEM with regression coefficients. For the sake of readability, non-significant associations of control variables have been omitted. *** p≤.001; ** p≤.01, *p≤.05.

In consideration of hypothesis 1, a highly significant positive association was observed between mistrust and deficits in the ability to mentalize (β=.704, p≤.001). Accordingly, participants with higher epistemic mistrust showed a lower mentalizing ability. Additionally, there was a marginally significant negative association between epistemic trust and deficits in the ability to mentalize (β=-.103, p=.055). In contrast, the association between credulity and the ability to mentalize was not supported by the data, as the observed association was not statistically significant.

Hypothesis 2 was fully confirmed. Deficits in the ability to mentalize showed significant and positive associations with violence supporting attitudes (β=.175, p≤.001), the tendency towards conspiracy mentality (β=.345, p≤.001), and the endorsement of antisemitic attitudes (β=.179, p≤.001).

Lastly, hypothesis 3 was partially confirmed. Indirect effect coefficients are presented in **Table 3**. The associations regarding epistemic mistrust all showed significant results. Accordingly, there was an indirect positive association between epistemic mistrust and violence supporting attitudes, conspiracy mentality as well as antisemitic attitudes via deficits in the ability to mentalize. Furthermore, the indirect association between epistemic trust and conspiracy mentality via deficits in the ability to mentalize showed marginally significant results (p = .056), while for antisemitic and violent extremist attitudes, the indirect association failed to reach significance (both p = .095). All indirect pathways involving epistemic credulity reached no significance.

**Table 3:** Indirect effect coefficients.

Indirect pathway	Coefficient	p-value	Confidence interval	
			Lower	Upper
Pathways with trust as the IV				
ETMCQ-Trust → MZQ →GEE	-.018	.095	-.039	.003
ETMCQ-Trust → MZQ →CMQ	-.035	.056	-.072	.001
ETMCQ-Trust → MZQ →Antisemitic attitudes	-.018	.095	-.002	.042
Pathways with mistrust as the IV				
ETMCQ-Mistrust → MZQ →GEE	.123	.005**	.038	.209
ETMCQ-Mistrust → MZQ →CMQ	.243	≤.001***	.125	.360
ETMCQ Mistrust → MZQ →Antisemitic attitudes	.128	.004**	.211	.042
Pathways with credulity as the IV				
ETMCQ-Credulity → MZQ →GEE	.016	.515	-.032	.064
ETMCQ Credulity → MZQ →CMQ	.031	.504	-.060	.122
ETMCQ-Credulity → MZQ →Antisemitic attitudes	.016	.513	-.064	.032

ETMCQ, Epistemic Trust, Mistrust, and Credulity Questionnaire; MZQ, Mentalization Questionnaire; GEE, Violent Extremist Attitudes Scale; CMQ, Conspiracy Mentality Questionnaire.

***p≤.001; **p≤.01, *p≤.05.

The control variable mental disorder demonstrated significant associations with epistemic credulity (β=-.345, p≤.001), mistrust (β=-.293, p≤.001), and deficits in the ability to mentalize (β=-.105, p=.019). Therefore, participants who reported having experienced a mental disorder within the past year exhibited higher levels of epistemic credulity, mistrust and lower mentalizing ability. In contrast, gender was significantly associated with epistemic trust (β=-.245, p≤.001), antisemitic thinking (β=-.166, p≤.001), and violence supporting attitudes (β=.212, p≤.001). Accordingly, female participants exhibited higher levels of epistemic trust, reduced antisemitic thinking and reduced violence supporting attitudes.

## Discussion

This study investigated the relationship between levels of mistrusting, trusting and credulous epistemic stances towards socially transmitted information and conspiracy mentality, antisemitic and violence supporting attitudes in a German community sample via deficits in the ability to mentalize. Findings revealed indirect positive associations between epistemic mistrust and extremist attitudes via deficits in mentalizing, i.e., deficits in adequately hypothesizing and wondering about internal motivations for human behavior. Moreover, for conspiracy mentality, there was a marginally significant indirect effect of epistemic trust via mentalizing deficits. Presence of a mental disorder was only associated with epistemic stance (mistrust and credulity) and mentalizing, but not with antisemitic, conspiracy, or violence supporting attitudes. Moreover, female gender was associated with higher epistemic trust and with reduced antisemitic and violence supporting attitudes.

As hypothesized, increased epistemic mistrust and increased extremist attitudes were indirectly related to mentalizing deficits, a pattern that is compatible with the proposed model, although it does not provide confirmation given the cross-sectional design. These findings suggest that mentalizing deficits may represent one pathway linking epistemic mistrust to extremist attitudes. However, at this point, alternative explanations such as reciprocal relationships or shared underlying vulnerabilities cannot be ruled out. Mentalizing as an indirect path between epistemic mistrust and extremist thinking is broadly in line with previous research linking mentalizing with resilience against various aversive outcomes, as e.g. against violent behavior in spite of early maltreatment (49), and identifying mentalizing deficits as part of the pathway linking epistemic mistrust and paranoid distress (29). Thus, these findings could inspire future longitudinal or experimental research examining psychological processes that may serve as potential starting points for intervention, in order to strengthen the individuals` abilities to cope with aversive experiences [e.g. (50)]. Mentalizing may represent one such candidate as it is the main focus of a therapy named mentalization-based therapy (MBT). MBT provides interventions targeted to improve mentalizing, which can also be applied in more general consulting or educational programs [e.g. (51)]. Whether targeting mentalizing in the general population would translate into reduced extremist attitudes, however, remains to be tested in future research.

Our findings of a marginally significant negative relationship between epistemic trust and conspiracy mentality indirectly associated via mentalizing deficits in this study complement previous findings of our workgroup of a positive indirect association between epistemic trust and conspiracy mentality via paranoid distress (29): as an interpretation, we contemplated whether high epistemic trust may render the individual specifically vulnerable for paranoid distress in times of (social) threat due to their tendency to typically turn to others, and may as such contribute to individuals turning to conspiracy beliefs as a form of emotional regulation (29). On a similar note, Douglas et al. (52) suggested that people may be drawn to conspiracy theories when there’s a chance for them to satisfy psychological motives (e.g. desire to be understood and seen). Similarly, Brauner et al. (28) found positive correlations for all epistemic stances with conspiracy mentality. However, the correlation with epistemic trust was not robust to multiple comparisons corrections; and the relationship between credulity and conspiracy mentality was full mediated by epistemic mistrust. Taken together, the relationship between epistemic trust and conspiracy mentality should be further explored, as different study designs as well as mediating variables may play a role in the inconsistent pattern so far.

Our findings on the confounding variable presence of mental disorder are in line with previous clinical research on the associations between epistemic stance, reduced mentalizing and psychopathology [e.g. (39, 53)]: Mentally suffering individuals showed more epistemic mistrust and credulity, and more deficits in mentalizing. Due to early and/or current burdens, individuals are hypothesized to be less stable in their mentalizing capacity, tend towards more negative self-views and towards reduced or more negatively biased interpretation of others` behavior and intentions [e.g. (54)]. Improving patients´ mentalizing capacity to reduce the suffering stemming from inner and outer turmoil is an explicit goal of MBT, but is also assumed a central change mechanism in psychotherapies in general not limited to MBT (30, 55).

Importantly, in this study on a community sample, we did not find that the presence of a mental disorder was related to any of the extremist attitudes. In line, extremist views are not considered psychopathological per se. Rather, our findings provide grounds for further research on these concepts mainly applied in clinical understanding and intervening, on the question of whether they indeed have the potential to go beyond the clinical and may be targeted as a general psychological skill of resilience against extremist attitudes.

On a psychological level, the relevance of aversive affective states and attempts to regulate these for the occurrence of extremist attitudes have been underscored. For example, the belief in conspiracy theories may serve as an inner explanation of one’s own perception of not being treated fairly (28), allowing a seemingly consistent explanation and alleged sense of stability in the face of ambiguity and uncertainty (52), while lacking in adequate mentalization of the underlying feelings. When the capacity to mentalize is enhanced, individuals may be able to better understand and communicate their inner states (mentalizing the self), potentially reducing emotional outbursts and impulsive, and possibly destructive, behaviors that are driven by aversive emotions. Additionally, individuals may become more capable of adequately hypothesizing and remaining open and curious about the inner states of others (mentalizing others).

Considering both the discussion of the need of considering gender in research and gender gaps in extremist developments, we integrated gender into the model. Our results showed that female participants were less likely to support violent extremist attitudes compared to male participants. Further, higher levels of epistemic trust came along with reduced endorsement of antisemitic attitudes in female participants in this study. These findings seem partially in line with the literature pointing to gender differences [e.g. (4)]. Yet, the relations need to be further investigated. Recent research on antisemitism found that gender associations differed between antisemitism regarding “Jews qua Jews” and regarding Israel: Increased Israel-related antisemitism was related with female gender in this study, increased antisemitism against Jews qua Jews with male gender (10). The authors stated further, that while the overall levels of antisemitism did not differ much across gender in their study, the expression of antisemitism may look different between men and women.

The findings of this study should be interpreted considering several limitations. We used an exploratory, cross-sectional design. We aimed to explore possible relations between the concepts of epistemic trust, mistrust and credulity, mentalizing, and extremist attitudes in a German community sample. Due to the use of a cross-sectional design, causal inferences between variables are not possible. Importantly, the literature does not converge on a single directional model of the relationship between epistemic stance and mentalizing, and their relationship to extremist thinking or other variables relevant for mental and social well-being. Some studies model epistemic stance as a predictor and mentalizing as a mediator of subsequent outcomes (23, 56), while others model epistemic trust, mistrust, and credulity as mediating mechanisms between developmental or attachment-related variables and later outcomes [e.g (27, 57)] or as mediators of the association between mentalizing and outcomes such as emotion dysregulation, thereby placing mentalizing sequentially before epistemic stance (53). At the current stage of research, all of these directions are theoretically grounded within mentalizing and epistemic trust theory, which conceptualizes epistemic trust, mistrust and credulity, and mentalizing as closely co-developing stances and capacities rooted in early social-communicative experience (25, 26). The here applied specification of epistemic stance as the predictor and mentalizing as the mediator draws on the conceptualization of epistemic trust, mistrust and credulity as relatively stable communicational stances (25), whereas mentalizing, as outlined above, was conceptualized to constitute a more dynamic, situationally fluctuating process particularly under stress (25, 31, 32). It should be noted, however, that this conceptual distinction between a relatively stable communicational stance and a relatively dynamic mentalizing capacity is not captured well by the self-report measures used in the present study: both the ETMCQ (27, 39) and the MZQ (40) assess relatively stable tendencies rather than situational or momentary fluctuations. Our model specification therefore rests on a theoretical distinction between the two constructs that is not directly reflected in their operationalization within this study. Notably, the internal consistency of the mistrust scale was below the commonly recommended threshold of .7 (58). This introduces measurement error that limits the generalizability of findings. The relatively low internal consistency is likely due to its small number of items (three). Consequently, results involving this scale should be interpreted cautiously, particularly as the analyses are exploratory in nature. However, as low internal consistency typically attenuates observed relationships, the observed associations may represent conservative estimates and may really be stronger than what we detected. Future studies should prioritize improving the psychometric properties of a measure of epistemic mistrust. Furthermore, self-report questionnaire data is subjectively biased. This in addition to social desirability effects may have influenced participants’ responses especially concerning the sensitive topics addressed in this study. Generally, mean scores of the extremist attitudes were rather low (for means and SDs see **Table 2**). While these scores may be representative for a large part of the German population, generalization of the findings to groups with higher levels of extremist attitudes is not warranted and should be investigated in future studies. Also we have not investigated a representative German population sample, but rather a community sample of people who all subscribed to the online panel provider, with the potential for a self-selection bias common to online panels. A further limitation concerns the absence of an *a priori* power analysis for the structural equation model. The sample size was determined by the objectives of the larger research project from which the data were derived rather than by requirements of the present model. Although a subsequent RMSEA-based sensitivity analysis indicated that the available sample size provided adequate statistical power for the estimated model, this analysis was conducted after data collection and therefore cannot replace prospective sample size planning. Moreover, our measures used present with several additional limitations: as we only used a nonspecific measure of a tendency towards conspiracy mentality, endorsement of violence supporting attitudes in hypothetical cases, and only two items assessing endorsement of antisemitic attitudes, we cannot claim to have reliably and thoroughly assessed extremist attitudes in the general population. Consequently, several relevant aspects were not addressed, such as postcolonial antisemitism and actual belief in specific conspiracy theories, let alone a proneness to violent behavior. Similarly, to the limitation mentioned above regarding the measure of relatively stable tendencies of epistemic stance and mentalizing, this study overall did not consider potential differences between state (situational) and trait (dispositional) factors that could impact the findings, especially regarding the sensitivity of the investigated constructs to context and world events. In addition, some of the model fit indices showed values outside the target range when assessing the measurement model quality. However, as described by Awang (46), the indices should not be viewed as strict cut-offs but the model should be described by the overall representation. Accordingly, the measurement models were accepted after a thorough inspection. Nevertheless, the interpretation of the results should take into account that not all indices correspond to the cut-off values. These limitations suggest that results should be interpreted with caution and be used to inspire future research to improve our understanding of various aspects regarding the explored concepts. We additionally acknowledge that clinical treatment concepts and strategies can only reach so far on a broader societal scale and will need elaboration and adaptation. We use concepts stemming from individual psychology while remaining acutely aware of the limitations of an individual approach to a phenomenon so deeply woven into society. Nevertheless, we believe in its value to further the understanding of extremist thinking and feeling.

## Data Availability

The datasets presented in this article are not readily available because anonymized data cannot be made publicly available due to ethical restrictions. They can be made available upon reasonable request for the purpose of scientific transparency. Requests to access the datasets should be directed to sophie.hauschild@med.uni-heidelberg.de.
